# The performance of directional area scattering factor proxies for correcting canopy structural and soil effects on leaf chlorophyll content estimation varies with canopy conditions in agricultural systems

**DOI:** 10.3389/fpls.2026.1884277

**Published:** 2026-09-30

**Authors:** Ruisi Bao, Xiaoyu Zhu, Ziwei Wang, Mengdi Wang, Liwen Fang, Lei Ding, Ziwei Zhang, Zhenwang Li, Changwei Tan, Jun Zhu

**Affiliations:** 1 Jiangsu Key Laboratory of Crop Genetics and Physiology/Jiangsu Key Laboratory of Crop Cultivation and Physiology, Agricultural College, Yangzhou University, Yangzhou, China; 2 Agro-Environmental Protection Institute, Ministry of Agriculture and Rural Affairs, Tianjin, China; 3 Schoolof Geomatics, Zhejiang University of Water Resources and Electric Power, Hangzhou, China; 4 School of Computer and Software, Nanjing University of Industry Technology, Nanjing, China

**Keywords:** canopy structure, global sensitivity analysis, leaf chlorophyll content, radiative transfer model, remote sensing

## Abstract

Accurate leaf chlorophyll content (LCC) estimation from canopy reflectance is essential for agricultural monitoring but remains challenging due to confounding effects of canopy structure and soil background. While spectral invariants theory, implemented through directional area scattering factor (DASF) proxies, offers a framework to decouple these effects from canopy bidirectional reflectance factor (BRF), their comparative performance across diverse canopy conditions remains unexamined. In this study, we comprehensively evaluated four DASF proxies, practical DASF (pDASF), improved DASF (iDASF), near-infrared reflectance of vegetation (NIRv), and NIRv from hyperspectral measurements (NIRVH2), for mitigating structural and soil effects on BRF and improving LCC retrieval. Using PROSAIL-5 simulations with global sensitivity analysis, we quantified the capacity of DASF-corrected BRFs to enhance LCC sensitivity while suppressing confounding factors. We then evaluated their applicability for LCC estimation under diverse canopy conditions using ground hyperspectral and simulated Sentinel-2 MSI data. DASF-corrected BRFs significantly enhanced spectral sensitivity to LCC while minimizing structural and background interference. This correction consistently outperformed uncorrected BRF in LCC estimation, reducing RMSE by 10.90%–15.88% (BRF/pDASF), 0.38%–11.62% (BRF/iDASF), 6.05%–16.47% (BRF/NIRv), and 7.44%–16.62% (BRF/NIRvH2). Notably, NIRv and NIRvH2 demonstrated relatively better estimation performance than pDASF and iDASF in sparse canopies (LAI < 2 m²/m²), whereas pDASF performed reliably in dense canopies (LAI > 3 m²/m²). This study reveals the differential applicability of DASF proxies across canopy conditions and sensor configurations, thereby advancing operational LCC retrieval and offering a robust framework for non-invasive assessment of physiological and biochemical traits in agricultural systems.

## 1 Introduction

Chlorophyll is the primary pigment driving solar energy conversion into chemical energy through light absorption and carboxylation reactions (Anderson, 1986; Richardson et al., 2002). As leaf chlorophyll content (LCC) can quantitatively reflect plant photosynthetic capacity, it has emerged as a key biochemical parameter for both leaf-level productivity assessment and ecosystem-scale carbon cycle modeling (Croft et al., 2017; Luo et al., 2018; Cai et al., 2021). In addition, LCC is a key agronomic parameter strongly linked to nitrogen status and senescence dynamics, it is therefore essential for monitoring vegetation health, guiding fertilizer applications, and optimizing field management practices (Richardson et al., 2002; Li et al., 2020). Traditional destructive analysis of LCC is laborious and time consuming. Remote sensing offers an efficient approach to non-destructively retrieve LCC at both leaf and canopy scale (Schlemmer et al., 2013).

Remote sensing estimation of LCC primarily employs three categories of approaches: empirical, physically-based, and hybrid methods (Verrelst et al., 2015; Berger et al., 2020). Empirical techniques provide a direct solution for estimating LCC based on statistical relationships between chlorophyll-sensitive reflectance bands and LCC. Physically-based methods, mainly implemented using radiative transfer models (RTM), mechanistically simulate interactions between leaf biochemistry, canopy structure, and canopy reflectance. Hybrid methods typically use RTM-simulated datasets to train machine learning algorithms, enabling accurate LCC retrieval while balancing physical interpretability and computational efficiency (Verrelst et al., 2015; Berger et al., 2020). Estimating LCC from leaf reflectance is relatively straightforward. However, deriving LCC from canopy reflectance remains challenging due to the confounding factors, including canopy structures (e.g., leaf area index, leaf angle distribution). Moreover, the coupled variation of canopy structure and LCC during plant growth further obscures LCC signals (Yu et al., 2014; Liu et al., 2023; Thorp et al., 2024; Li et al., 2026). These complications hinder the remote retrieval of LCC across growth seasons and regions (Ollinger, 2011). Furthermore, soil background interference (e.g., soil type, color, and moisture conditions) dominates spectral variance during early growth stages (Gilabert et al., 2002; Yang, 2022; Yang et al., 2025), and the influence diminishes as vegetation grows and ground cover increases. These challenges necessitate advanced inversion methods that remove or mitigate the influence of canopy structure and soil background in the LCC estimation.

To date, a variety of approaches have been developed to mitigate the confounding effects of canopy structure and soil background effects on canopy-scale LCC estimation. The first method applies spectral transformations to canopy reflectance, including derivative transformation, continuum removal, and continuous wavelet analysis. These techniques reduce soil and atmospheric noise while improving spectral sensitivity to LCC (Reum and Zhang, 2007; Li et al., 2018; Nagy et al., 2024). The second method is designing enhanced vegetation indices (VIs) that are sensitive to LCC but resistant to confounding factors, such as the LAI-insensitive chlorophyll index (Li et al., 2020), angular insensitivity vegetation index (He et al., 2016), and chlorophyll sensitive index (Zhang et al., 2022). In addition, several VI matrixes that combine LCC-sensitive VI with LAI-sensitive VI (via division or subtraction) were also introduced to alleviate the canopy structural effect on LCC estimation, such as the ratio of the transformed chlorophyll in reflectance index (TCARI) to the optimized soil-adjusted vegetation index (OSAVI) (Wu et al., 2008). The third method is employing the physically-based two-step inversion algorithm (Croft et al., 2020; Li et al., 2021). This approach firstly converts canopy reflectance to leaf reflectance through canopy RTMs, then the simulated leaf reflectance was used to estimate LCC based on leaf optical models or machine learning algorithms. However, a common drawback of these methods is their inability to fully separate canopy structural effects from leaf biochemical signals, largely because they rely on empirical relationships or indirect parameterizations that do not explicitly account for the radiative transfer processes within the canopy. As a result, the confounding influences of canopy structure, particularly leaf area index and leaf angle distribution, on reflectance spectra remain inadequately resolved, leading to compromised LCC retrieval accuracy, especially under varying canopy conditions.

In this context, spectral invariant theory offers a physically based and theoretically rigorous framework to overcome these limitations (Huang et al., 2007; Knyazikhin et al., 2013). The theory characterizes canopy bidirectional reflectance factor (BRF) as a function of wavelength-dependent leaf single scattering albedo and wavelength-invariant structural parameters (Knyazikhin et al., 2013; Zeng et al., 2018), with the directional area scattering factor (DASF) serving as a key structural parameter for correcting canopy structural effects and estimating canopy scattering coefficients (CSC), which are directly related to leaf optical properties. However, since DASF cannot be directly retrieved from remote sensing data, several proxies have been developed. These proxies can be broadly grouped into two categories. The first includes radiative-transfer-based proxies: pDASF (Knyazikhin et al., 2013) and iDASF (Lin et al., 2023), which differ primarily in whether LDM variability is considered. The second comprises spectral-index-based proxies: NIRv (Badgley et al., 2017) and NIRv from Hyperspectral measurements (NIRvH) (Zeng et al., 2021), with NIRvH designed to minimize residual soil effects identified in NIRv. While these DASF proxies have shown potential in mitigating structural effects in biophysical variable retrieval (Li and Liang, 2023; Liu et al., 2023; Wan et al., 2024), their performance in LCC estimation under varying canopy conditions and across different sensor types remains inadequately investigated. Specifically, there is a lack of systematic comparative studies evaluating the efficacy of pDASF, iDASF, NIRv, and NIRvH for LCC estimation across diverse canopy structures and soil backgrounds, especially during critical early growth stages of herbaceous crops. Moreover, while NIRv and pDASF were originally designed to be compatible with broadband multispectral data, NIRvH2 and iDASF rely on spectral features that require hyperspectral or narrow-band observations. Their applicability to widely used broadband satellite platforms (e.g., Landsat-8, Sentinel-2) for operational LCC monitoring is still uncertain.

The objective of this study was to comprehensively evaluate the four DASF estimation methods (pDASF, iDASF, NIRv, and NIRvH) in correcting the confounding effect on canopy BRF to improve LCC estimation using RTM simulations, ground measurements, and simulated satellite data. The specific aims are (1) to explore the sensitivity of DASF-corrected BRFs (CSCs) to LCC, canopy structural parameters (LAI and ALA), and soil background (soil type, brightness, and moisture); (2) to improve LCC estimation by incorporating DASF to reduce canopy structural and soil background effects on canopy BRF; and (3) to examine the applicability of the four DASF estimation methods for ground hyperspectral and simulated Sentinel-2 MSI data and their performance for LCC estimation across varying canopy conditions.

## 2 Materials and methods

### 2.1 Field datasets

To develop and validate LCC estimation models using canopy BRF and CSCs, we collected a wide range of ground measurements from two publicly available datasets: the GFZ Data Services (https://dataservices.gfz-potsdam.de/) and Ecological Spectral Information System (EcoSIS) (https://ecosis.org/). A total of 753 full range canopy spectral measurements and corresponding LCC data were collected from two distinct sites, Karlsruhe and Neusling. The LCC measurements covered two vegetation types: grassland and cropland, the details are listed in **Table 1**.

**Table 1:** Detailed information on the field datasets.

Dataset	Time	Vegetation type	Number of samples	Canopy spectral method	LCC method	Citation
Karlsruhe	2016-2017	Grassland	556	ASD FieldSpec	PROSPECT inversion	(Kattenborn et al., 2019)
Neusling	2011-2012	Cropland	197	Airborne images	SPAD	(Hank et al., 2015, 2016; Cherif et al., 2023)

The Karlsruhe dataset was collected from the Karlsruhe Institute of Technology, Germany in 2016 and 2017 (Kattenborn et al., 2019), 38 grassland species belonging to different plant functional types were cultivated outdoor in four repetitions in separate pots with a size of 0.4 m × 0.4 m, the data were measured on a weekly basis. Canopy BRF within each plot was measured using an ASD FieldSpec III (ASD, Inc. Boulder, CO, USA) spectrometer across the 350–2500 nm range. LCC were derived using an inversion of leaf spectra and the PROSPECT-D model with normalized root mean square error of 7.91%. LAI was collected using an Accu-PAR LP-80 ceptometer, detailed description of the field measurements can be found in (Kattenborn et al., 2019).

The collection of Neusling dataset that contains airborne hyperspectral data as well as accompanying *in-situ* data was acquired in 2011 and 2012 in the Neusling region of Southern Germany (Hank et al., 2015; Locherer et al., 2015; Hank et al., 2016). The region is dominated by intensive agricultural cultivation with the most common crops are winter wheat, maize, sugar beet, winter barley, and rapeseed. The airborne hyperspectral images were collected using APEX, AVIS, and HySpex instrument with spectral bands ranging from visible to shortwave infrared (SWIR) and spectral resolution of 2.8-6.6 nm, the spectral data were then resampled to a 400–2500 nm range with a 1 nm resolution using forward and backward linear interpolation (Cherif et al., 2023). LAI was measured using LI-COR LAI-2000 plant canopy analyzers (LI-COR, Inc., Nebraska, USA), and leaf chlorophyll was sampled using a Konica-Minolta SPAD-502plus instrument, which was then converted into LCC using Equation developed by (Lichtenthaler, 1987). See (Locherer et al., 2015) and (Cherif et al., 2023) for more information.

### 2.2 PROSAIL-5 model

This study employed PROSAIL-5 RTM (Jacquemoud et al., 2009) to simulate canopy BRF across diverse vegetation growth scenarios. The PROSAIL-5 model was developed based on the coupled PROSPECT-5 leaf optical properties model (Feret et al., 2008) and the 4SAIL canopy bidirectional reflectance model (Verhoef, 1984). PROSAIL-5 simulates nadir-viewed canopy BRF (from 400 to 2500 nm at 1 nm resolution), assuming negligible atmospheric effect. For this study, the PROSAIL-5 model bindings implemented in Python by Domenzain et al. (2019) were employed. The ranges of the main input variables for the PROSAIL simulations of herbaceous-like canopies were determined based on site-specific data collected during field campaigns and supplemented by relevant published literature (Berger et al., 2020; Gao et al., 2022; Bhadra et al., 2024). The ranges considered for our study are listed in **Table 2**.

**Table 2:** Input parameters for PROSPECT-5 and SAIL model and the value ranges of the nine parameters (in bold) in the global sensitivity analysis.

Model	Variable	Symbol	Unit	Range
PROSPECT-5	**Leaf structure parameter**	N	–	1.0-2.5
	**Leaf chlorophyll content**	LCC	μg cm^-^²	5-80
	**Equivalent water thickness**	C_w_	g cm^-^²	0.002-0.03
	**Dry matter content**	C_m_	g cm^-^²	0.01-0.02
	Leaf carotenoid content	Car	μg cm^-^²	8
	brown pigment	C_brown_	μg cm^-^²	0
SAIL	**Leaf area index**	LAI	m² m^-^²	0.5-8
	**Average leaf inclination angle**	ALA	°	5-80
	Hotspot	hot	–	0.2
	**Dry/Wet soil factor**	psoil	–	0-1
	**Soil brightness factor**	rsoil	–	0-1
	**Solar zenith angle**	θ_s_	°	10-50
	Observer zenith angle	θ_v_	°	0
	Relative azimuth angle	φ	°	0

### 2.3 Simulation of Senteinel-2A MSI data

To evaluate the applicability and performance of the four DASF proxies to correct canopy structural effect in LCC estimation for broadband satellite data, the observed hyperspectral data were used to simulate Sentinel-2A MSI data by means of a spectral convolution of the hyperspectral data with the spectral response functions (SRF) of the respective sensors. SRF of Sentinel-2A MSI were obtained from Sentinel portal (https://sentinels.copernicus.eu/web/sentinel/copernicus/sentinel-2), as illustrated in **Figure 1**. The convolution equation is presented as follows (Equation 1):

**Figure 1:**
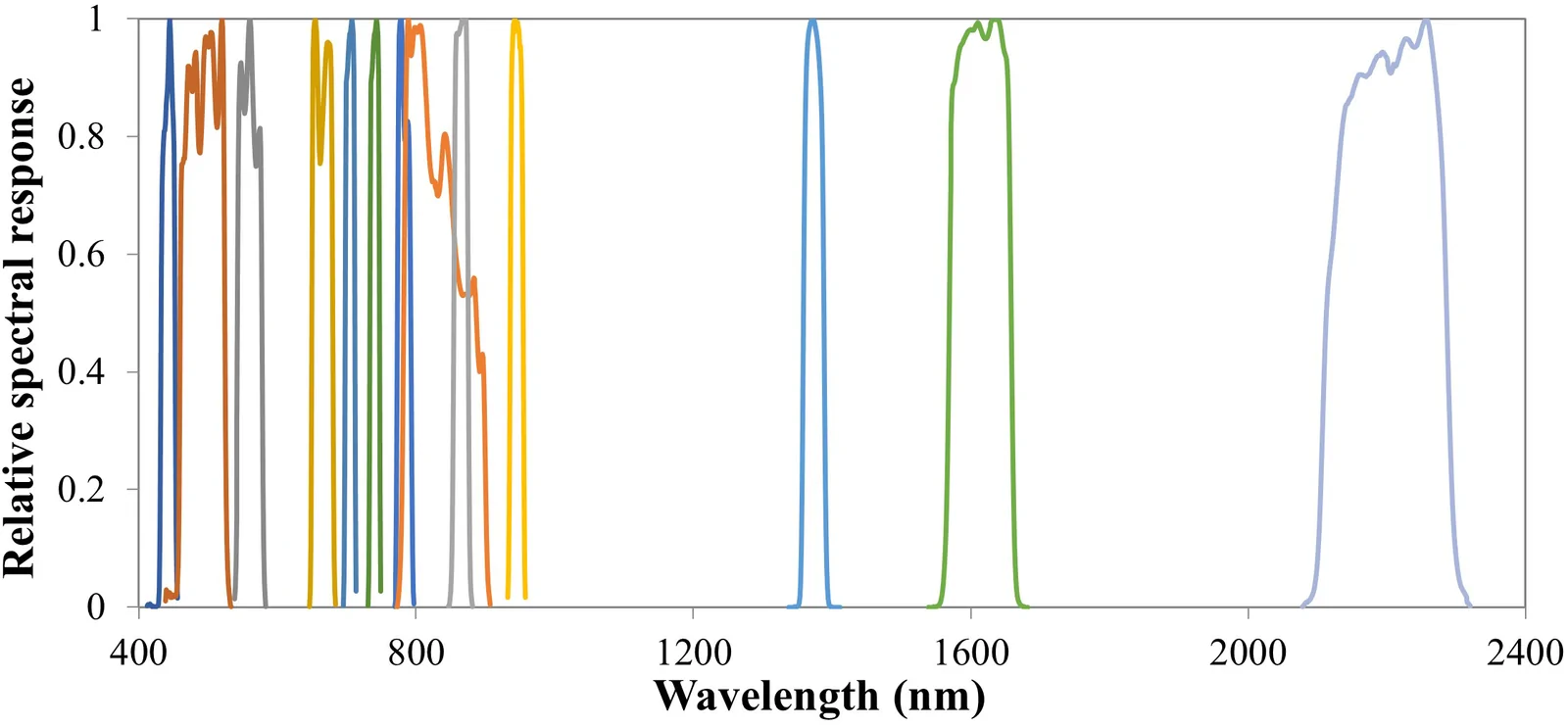
Spectral response function of Sentinel-2A MSI.

(1) $$\rho_{band}=\frac{\int_{\lambda_{1}}^{\lambda_{2}}\rho_{\lambda }\times SRF_{\lambda }d\lambda }{\int_{\lambda_{1}}^{\lambda_{2}}SRF_{\lambda }d\lambda }$$

where, $\rho_{band}$ is the BRF of the simulated band, $\rho_{\lambda }$ is the BRF value of the measured spectrum. $\lambda_{1}$ and $\lambda_{2}$ are the corresponding wavelengths at both ends of the remote sensing band. $SRF_{\lambda }$ is the spectral response function of the corresponding sensor.

### 2.4 Estimation of DASF and CSC from canopy BRF

If the impact of canopy background on canopy BRF is negligible, BRF of a vegetation canopy can be parameterized by leaf single scattering spectral albedo ( $\omega_{\lambda }$) and three spectrally invariant variables, namely canopy interceptance ( $i_{0}$), recollision probability (p), and directional escape density ( ρ(Ω)) (Huang et al., 2007; Knyazikhin et al., 2013) (Equation 2):

(2) $$BRF_{\lambda }(\text{Ω})=\frac{\rho (\text{Ω})i_{0}}{1-\omega_{\lambda }p}\omega_{\lambda }$$

where $BRF_{\lambda }(\text{Ω})$ is canopy BRF in a given direction Ω at the specific wavelength. By combining the three spectral-invariant variables (p, $i_{0}$, ρ(Ω)), DASF can be combined as (Equation 3):

(3) $$DASF=\frac{\rho (\text{Ω})i_{0}}{1-p}$$

As a fundamental structural parameter, DASF can be used to minimize structural influences from canopy BRF and derive CSC (Equation 4), which exhibiting stronger correlations to leaf biochemical concentrations compared to BRF (Knyazikhin et al., 2013).

(4) $$CSC_{\lambda }=\frac{BRF_{\lambda }(\text{Ω})}{DASF}$$

Four DASF proxies were selected in this study to correct structural and soil background effects on canopy BRF. These proxies fall into two complementary categories: radiative-transfer-based proxies (pDASF and iDASF), grounded in spectral invariant theory with strong physical interpretability, and spectral-index-based proxies (NIRv and NIRvH2), which are computationally efficient and widely applied. pDASF is the most classic proxy, serving as the foundation for subsequent developments, while iDASF improves upon it by incorporating leaf dry matter variability. NIRv and NIRvH2 are simpler to compute and have been extensively used in agricultural remote sensing. Systematic comparison of these two categories allows us to evaluate their respective strengths and to identify their optimal application scenarios across canopy conditions and sensor configurations, thereby providing practical guidance for LCC estimation in agriculture.

#### 2.4.1 Estimation of pDASF

Knyazikhin et al. (2013) proposed pDASF to characterize the canopy structure of dense vegetation. Under negligible soil background conditions, pDASF can be directly derived from the BRF spectrum in the 710–790 nm range without requiring prior knowledge of leaf albedo. The key step to retrieve pDASF is to calculate the reference leaf albedo ( $\omega_{0\lambda }$) using the PROSPECT-5 simulation with N, LCC, Cw, and Cm set to 1.5, 16 μg/cm², 0.005 g/cm², and 0.002 g/cm², respectively. Then, the slope (k) and intercept (b) of the $BRF_{\lambda }(\text{Ω})/\omega_{0\lambda }$ versus $BRF_{\lambda }(\text{Ω})$relationship are derived. Finally, pDASF is calculated as b/(1-k).

#### 2.4.2 Estimation of iDASF

Lin et al. (2023) proposed the improved DASF (iDASF) to account for variations in LDM. Compared to pDASF described in Section 2.4.1, iDASF involves one more step for estimating E, which denotes the bias produced by LDM variation in estimating pDASF. iDASF can be expressed as (Equation 5):

(5) $$iDASF=\frac{b}{1-k-E}$$

where k and b are the slope and intercept of the $BRF_{\lambda }(\text{Ω})/\omega_{0\lambda }$ versus $BRF_{\lambda }(\text{Ω})$ relationship as described in Section 2.4.1, E is the correcting factor that can be modelled as Lin et al. (2023) (Equation 6):

(6) $$E=\exp(8.2475\times BRF_{710}-10.1829\times BRF_{2260}-\;3.6817)-0.0272$$

where $BRF_{710}$ and $BRF_{2260}$ is the canopy BRF at 710 nm and 2260 nm.

#### 2.4.3 Estimation of NIRv

NIRv is mathematically expressed as the product of NIR BRF and NDVI (Badgley et al., 2017), calculated as (Equation 7):

(7) $$NIRv\;=\;NDVI\;\times BRF_{NIR}$$

where NDVI is calculated based on red (630–690 nm) and NIR BRF (750–900 nm) and NIR is the BRF in the NIR region.

#### 2.4.4 Estimation of NIRvH2

To further minimize the residual soil background contributions on NIRv, Zeng et al. (2021) proposed NIRvH2. NIRvH2 takes advantage of the spectral shape variations in the red-edge region to minimize the soil effect (Equations 8 and 9).

(8) $$NIRvH2=BRF_{NIR}-BRF_{R}-\;k\times (\lambda_{NIR}-\;\lambda_{R})\;$$

(9) $$k=(BRF_{LS1}-BRF_{LS2})/(\lambda_{LS1}-\;\lambda_{LS2})\;$$

where $BRF_{NIR}$ and $BRF_{R}$ are the BRF in the red (678 nm) and NIR region, $BRF_{LS1}$ and $BRF_{LS2}$ represent BRF in either one of the two leaf spectral-invariant regions: red band (675–681 nm) or NIR band (778–800 nm), respectively, according to Zeng et al. (2021), $\lambda_{R}$, $\lambda_{NIR}$, $\lambda_{LS1}$, and $\lambda_{LS2}$ are the wavelengths of the bands used in the formulation.

### 2.5 Random forest model

The random forest (RF) regression model was used to establish the empirical relationship between LCC and canopy BRF or CSCs for LCC estimation. The RF algorithm constructs an ensemble of decision trees, with each tree trained on randomly selected subsets of the total training dataset (Breiman, 2001), final predictions are generated by averaging outputs from all individual trees. The RF was selected for its ability to model complex, nonlinear relationships between spectral features and LCC without requiring additional assumptions about data distribution. Furthermore, its inherent resistance to overfitting and its provision of feature importance metrics offer valuable insights for interpretation (Belgiu and Drăguţ, 2016). To optimize the RF model, a grid search with repeated 5-fold cross-validation was performed over the search ranges of two key hyperparameters: *ntree* (100–1000) and *mtry* (2 to total predictors). Finally, the hyperparameter combination yielding the lowest average root mean square error (RMSE) across validation folds was selected, with *ntree* were set to 500 and *mtry* was set to one-third of the total variables.

To identify the most important spectral bands for LCC prediction, the relative importance of each spectral band was assessed using the Boruta algorithm (Kursa and Rudnicki, 2010). This analysis employed 500 maximum random forest iterations with 0.99 confidence level, utilizing z-scores from mean decrease accuracy to derive permutation importance. Implementation was conducted using the “Boruta” packages within the statistical software R 3.6.2. To reduce the dimension of the input hyperspectral data for the RF model, the Pearson correlation coefficient (R) was used to analyze the relationship between the LCC and each band of canopy BRF or CSCs, only the VIS and NIR wavebands (500–1100 nm) were used in the estimation model due to the high sensitivity of the spectral bands to LCC.

### 2.6 Model simulations and evaluation

#### 2.6.1 Sensitivity analysis

A sensitivity analysis was performed to investigate the linkages between canopy BRF (or CSCs), LCC, canopy structure, and soil background using the PROSAIL-5 model. We firstly analyzed the sensitivity of simulated BRF and CSCs to variations in LCC, ALA, LAI, soil type, soil brightness, and soil moisture. This was done by altering one parameter at a time within the model while keeping all other parameters fixed. We also examined the relationships between pDASF and the other there DASF proxies using PROSAIL-5 simulations with all parameters were randomly varied within the predefined ranges.

To investigate the spectral response of LCC to canopy BRF and CSCs under different canopy background scenarios, we used the Sobol’ global sensitivity analysis (GSA) algorithm (Sobol', 2001) that implemented in the Python ‘SALib’ package (Herman and Usher, 2017) to conduct GSA on the PROSAIL-5 simulated datasets. The Sobol’ method is a variance-based GSA approach, which is particularly effective in quantifying both main sensitivity effect (Si, or first-order effect), which represent the individual contribution of each input parameter to the model output, and total sensitivity effect (STi), which encompass the first-order effect along with interactions between input variables, thereby capturing the overall contribution of each parameter to the model output (Song et al., 2012). The detail information on the Sobol’ GAS method can be seen in the **Supplementary Material**.

Five scenarios were designed for GSA with three focused on soil background variations (soil brightness (rsoil, ranging from 0 to 1 with the step of 0.1), soil type, and soil moisture), and two examined canopy structure variations (including 16 levels of LAI ranging from 0.5 to 8 in increments of 0.5 and 16 levels of ALA ranging from 5° to 80° in increments of 5°) (**Table 3**). Soil spectral reflectance representing various soil types and soil moisture were obtained from two public datasets (Liu et al., 2002; Lobell and Asner, 2002), the detail information can be found in the **Supplementary Material** (**Supplementary Figure S1**). The influence of the canopy structure and soil background on the contribution of LCC to canopy BRF was examined by repeatedly running GSA for each of the 14 soil moisture contents, 31 soil types, 11 soil brightness, 16 LAI and 16 ALA, the mean first-order sensitivity index (Si) from GSA of each scenario was used to quantify the contribution (sensitivity) of LCC to spectral bands. Previous studies recommended 20000–40000 PROSAIL-5 simulations can ensure stable GSA results (Morcillo-Pallarés et al., 2019; Gao et al., 2022), we randomly sampled 20480 PROSAIL-5 parameter combinations within predefined ranges using Sobol’ sequences, generating corresponding canopy spectra through forward modeling.

**Table 3:** Design of global sensitivity analysis (GSA) scenarios using the PROSAIL-5 model.

Scenario	Objective	Varied parameter	Levels/range	Other parameters per level	GSA iterations per scenario
S1	Assess effect of soil background	Soil brightness	11 levels (0 to 1, step = 0.1)	Randomly sampled using Sobol’ sequences (20480 combinations)	11 × 20480 simulations
S2	Assess effect of soil background	Soil type	31 soil types		31 × 20480 simulations
S3	Assess effect of soil background	Soil moisture content	14 levels		14 × 20480 simulations
S4	Assess effect of canopy structure	LAI	16 levels (0.5 to 8, step = 0.5)		16 × 20480 simulations
S5	Assess effect of canopy structure	ALA	16 levels (5° to 80°, step = 5°)		16 × 20480 simulations

#### 2.6.2 Model evaluation

To test the performance of canopy BRF and CSCs for estimating LCC, the empirical RF models established with ground hyperspectral and simulated satellite data were validated using the 5-fold cross validation method. The procedure divides the datasets into the training dataset (80%) and the testing dataset (20%), and the RF model repeated ran 5 times in order to obtain estimates for all samples (Hastie et al., 2009; Yates et al., 2023). Model performance was quantified using the coefficient of determination (R²), RMSE, and mean absolute error (MAE) based on comparisons between measured and estimated LCC.

In this study, LAI served as an indicator of canopy structure. To evaluate the performance of DASF in mitigating the influence of canopy structure on canopy BRF across varied canopy conditions, we also compared the RF model performance in estimating LCC using BRF and CSCs under different LAI levels to highlight their differences.

## 3 Results

### 3.1 Influence of LCC, canopy structure, and soil background on canopy BRF and CSCs using PROSAIL simulations

**Figure 2** illustrates PROSAIL-simulated canopy BRF and CSCs across varying LCC, canopy structure, and soil background. Generally, it was observed that correcting BRF by DASF could effectively minimize structural and soil-induced spectral variations while amplifying LCC-related spectral sensitivity. Compared to canopy BRF, CSCs demonstrated marginally enhanced LCC sensitivity in the visible spectrum (400–800 nm) and substantially greater sensitivity in NIR and SWIR regions (**Figures 2A–E**). This enhancement was particularly pronounced for BRF/NIRv and BRF/NIRvH2 at low LCC levels (<10 μg/cm²). With increased LCC, pDASF and iDASF showed an increasing trend, whereas NIRv and NIRvH2 increased firstly and then kept stable (LCC > 40 μg/cm²) (**Figure 2F**). Canopy BRF was sensitive to ALA variations within the full spectral region (**Figure 2G**), which could be substantially reduced by DASF corrections except for ALA = 80°, but BRF/iDASF retained slightly higher ALA sensitivity than other corrected BRFs (**Figures 2H–K**). The four DASF proxies all showed a decreasing trend with ALA increase, while a smaller decreasing trend was observed for BRF/NIRv and BRF/NIRvH2 (**Figure 2L**). Canopy BRF showed strong LAI dependence in red, NIR, and SWIR regions (**Figure 2M**). DASF corrections can effectively reduce this sensitivity for LAI >2.0 m²/m², though residual susceptibility persisted at lower LAI values (**Figures 2N–Q**). With increased LAI, the four DASF proxies firstly increase and then kept stable (LAI > 4.0 m²/m²), while the increase of BRF/NIRv and BRF/NIRvH2 was slower (**Figure 2R**).

**Figure 2:**
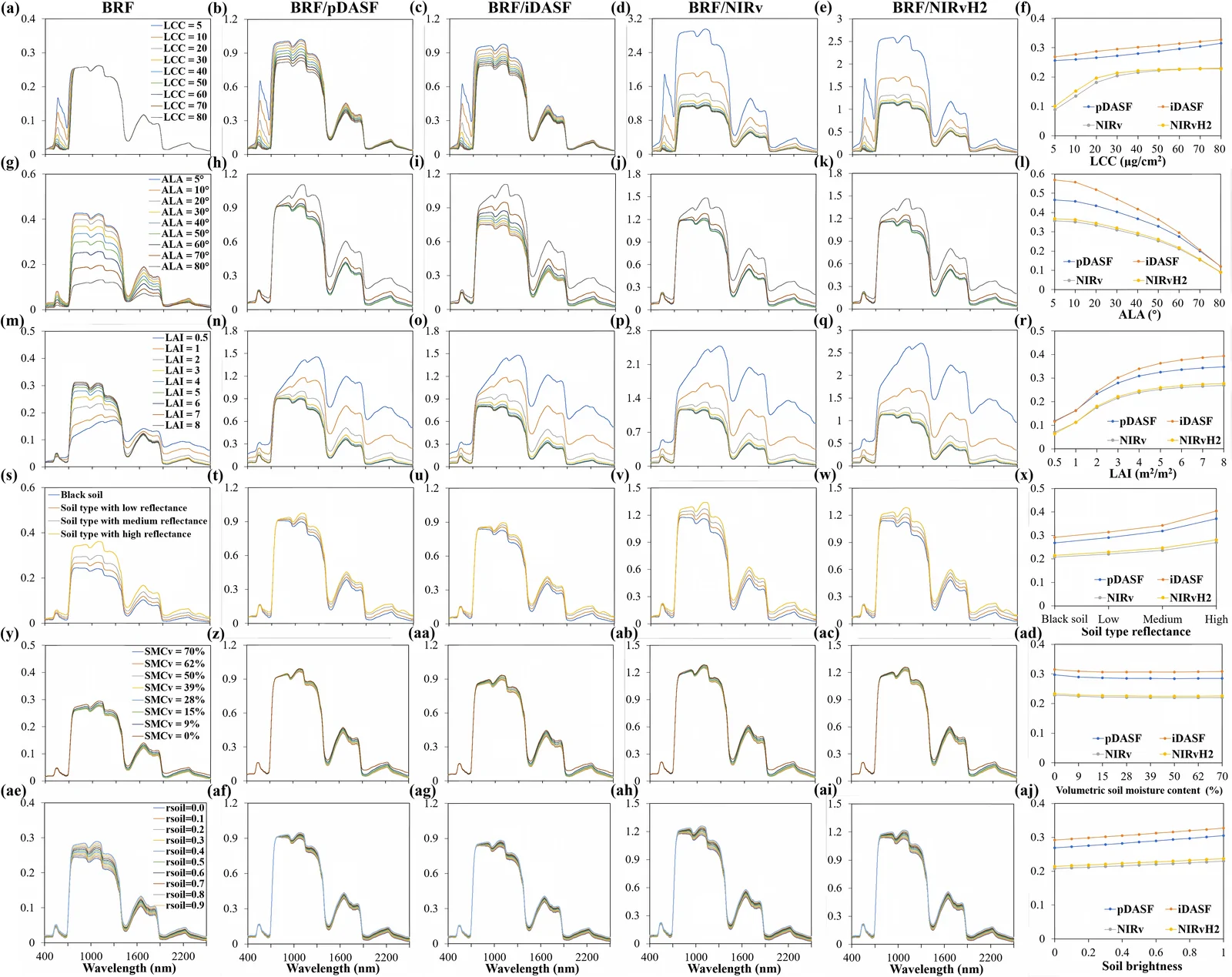
Simulated sensitivities of BRF, BRF/pDASF, BRF/iDASF, BRF/NIRv, and BRF/NIRvH2, and four DASFs to the variations in LCC **(A–F)**, ALA **(G–L)**, LAI **(M–R)**, soil type **(S–X)**, soil moisture content **(Y-AD)**, and soil brightness **(AE-AJ)** using PROSAIL-5 model. For each simulation, only one parameter was changed with the other parameters default. The simulated BRF and DASF-corrected BRFs for soil background variation **(D–F)** were under LAI = 2.0 m²/m². SMCv represents volumetric soil moisture content.

The influence of soil background on canopy BRF is highly dependent on the LAI conditions (**Figures 2S-aj**; **Supplementary Figure S2–S6**). For sparsely vegetated canopies (LAI < 3.0 m²/m²), soil background significantly influenced canopy BRF (**Figures 2S, Y, AE**; **Supplementary Figure S2-S3**), though this was partially mitigated by DASF corrections, particularly in the red-edge region (690–745 nm). However, CSCs still exhibited substantial variation due to soil background changes in other spectral regions. In dense canopies with LAI between 3.0 m²/m² and 6.0 m²/m², soil influence on canopy BRF was greatly reduced, but residual effect of soil type and brightness still persisted in the red, NIR, and SWIR regions (**Supplementary Figures S4, S5**). While CSCs showed reduced soil sensitivity, subtle residual soil influence remained evident in the red and SWIR regions. For highly dense canopies (LAI > 6.0 m²/m²), soil effect on canopy BRF were negligible, with DASF corrections further suppressing soil-related variations, especially in NIR and SWIR regions (**Supplementary Figure S6**). Among the four DASF proxies, pDASF and iDASF were more susceptible to soil property variations (**Figures 2X, AD, AJ**; **Supplementary Figure S7**), while NIRvH2 demonstrated relatively greater resistance to soil background changes. All four DASF proxies exhibited an increasing trend with increased soil brightness and soil-type reflectance, though pDASF and iDASF increased more rapidly than NIRv and NIRvH2. This increasing trend slowed with higher LAI. Soil moisture primarily affected DASF proxies at LAI < 1.0 m²/m², pDASF and iDASF decreased firstly and then stabilized (volumetric soil moisture content > 15%), whereas NIRv and NIRvH2 remained stable across soil moisture gradients.

### 3.2 Canopy spectral response to LCC under canopy structure and soil background variation using global sensitive analysis

GSA reveals that LCC predominantly influenced canopy BRF within the 400–750 nm spectral range, with negligible contributions at longer wavelengths (**Supplementary Figure S8**). BRF/iDASF exhibited a similar LCC response with canopy BRF in the 400–750 nm range, though a weak response was also observed in NIR bands (**Supplementary Figure S8C**). In contrast, BRF/pDASF, BRF/NIRv, and BRF/NIRvH2 demonstrated extended LCC sensitivity across broader visible, NIR, and SWIR spectral regions. The primary LCC contribution peaks occurred at 640 nm and 691 nm for canopy BRF, shifting to 559 nm and 714–718 nm for CSCs.

Under ALA variation, the contributions of LCC to canopy BRF and CSCs were similar in the 400–750 nm region (**Figure 3A**). However, BRF/iDASF and BRF/NIRvH2 showed slightly reduced LCC sensitivity at the two peak positions, while BRF/pDASF and BRF/NIRv exhibited marginally enhanced sensitivity. Under LAI variations, DASF corrections significantly improved LCC sensitivity within the chlorophyll-sensitive region (400–750 nm) compared to canopy BRF (**Figure 3B**), with BRF/NIRv demonstrating the highest sensitivity, followed by BRF/pDASF and BRF/NIRvH2. Compared to canopy BRF, soil type variations enhanced BRF/pDASF sensitivity to LCC, particularly at the two peak positions (**Figure 3C**), while weakened BRF/NIRvH2 sensitivity in visible and red-edge regions, BRF/NIRv and BRF/iDASF maintained LCC sensitivity comparable to canopy BRF in these regions. Soil moisture variation enhanced LCC sensitivity for all CSCs at the two peak positions compared to canopy BRF (**Figure 3D**), the enhancement for BRF/pDASF and BRF/NIRv was larger than BRF/iDASF and BRF/NIRvH2. Soil brightness variations amplified BRF/pDASF and BRF/NIRv sensitivity to LCC at the two peak positions compared to canopy BRF, while contribute similarly to BRF/iDASF and BRF/NIRvH2 (**Figure 3E**).

**Figure 3:**
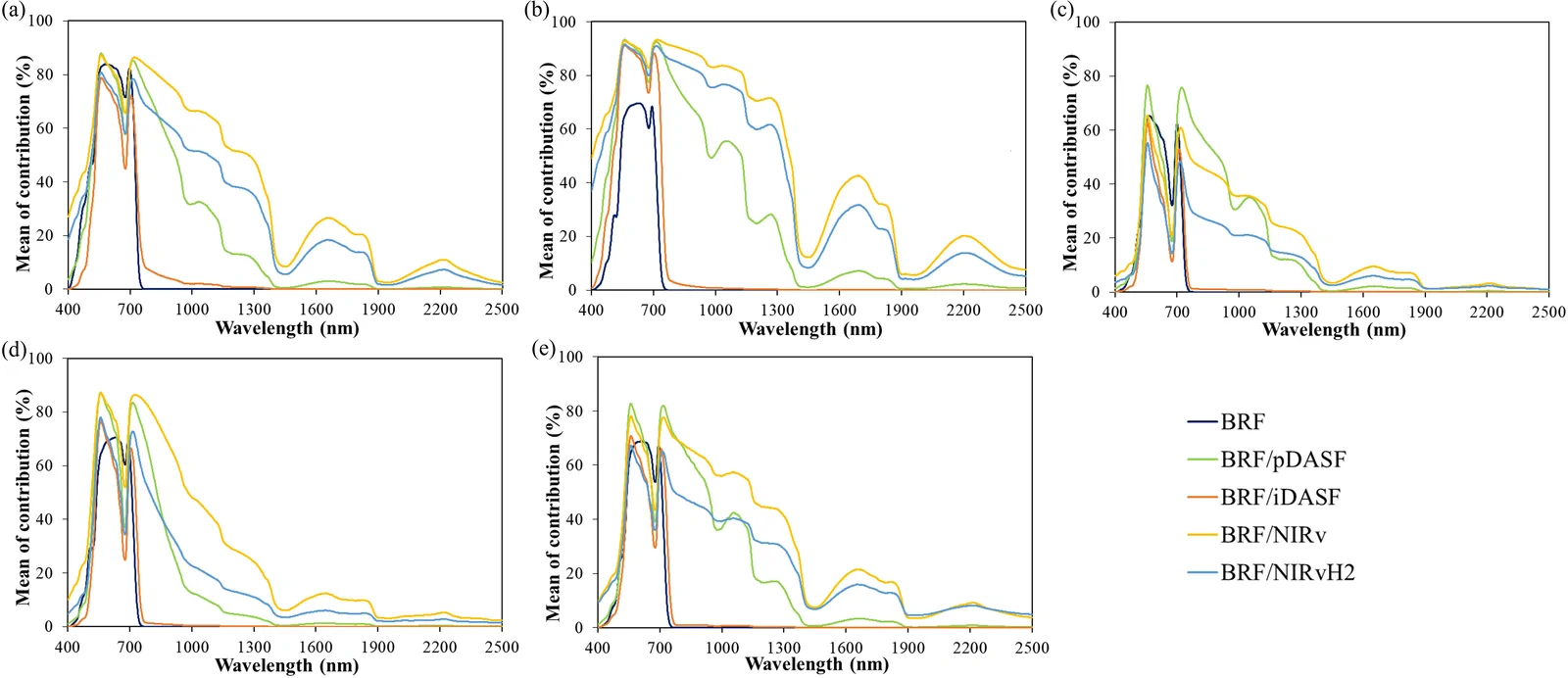
Mean first-order sensitivity indices of hyperspectral canopy BRF to LCC for variation in ALA **(A)**, LAI **(B)**, soil type **(C)**, soil moisture **(D)**, and soil brightness **(E)** using global sensitivity analysis.

### 3.3 DASF proxy interrelationships and their parameter sensitivity

**Figure 4** illustrates the relationships between pDASF and the other three DASF proxies derived from PROSAIL-5 simulations. NIRvH2 exhibited the strongest linear correlation with pDASF (R² = 0.935), whereas iDASF showed comparatively weaker relationship with pDASF (R² = 0.882). However, these relationships displayed notable LCC dependence, with reduced correlations observed at low chlorophyll levels (LCC < 30 μg/cm²). Further analysis through GSA quantified the contribution of PROSAIL-5 parameters to the four DASF proxies (**Figure 4D**). It was observed that the two canopy structural parameters (ALA and LAI) contributed the most to the four DASF proxies, followed by Cm and N. LCC significantly influenced NIRv and NIRvH2, exerted moderate effect on pDASF, and showed minimal impact on iDASF. Other parameters, such as Cw, TTs, Psoil and Rsoil produce negligible contributions to the four DASF proxies.

**Figure 4:**
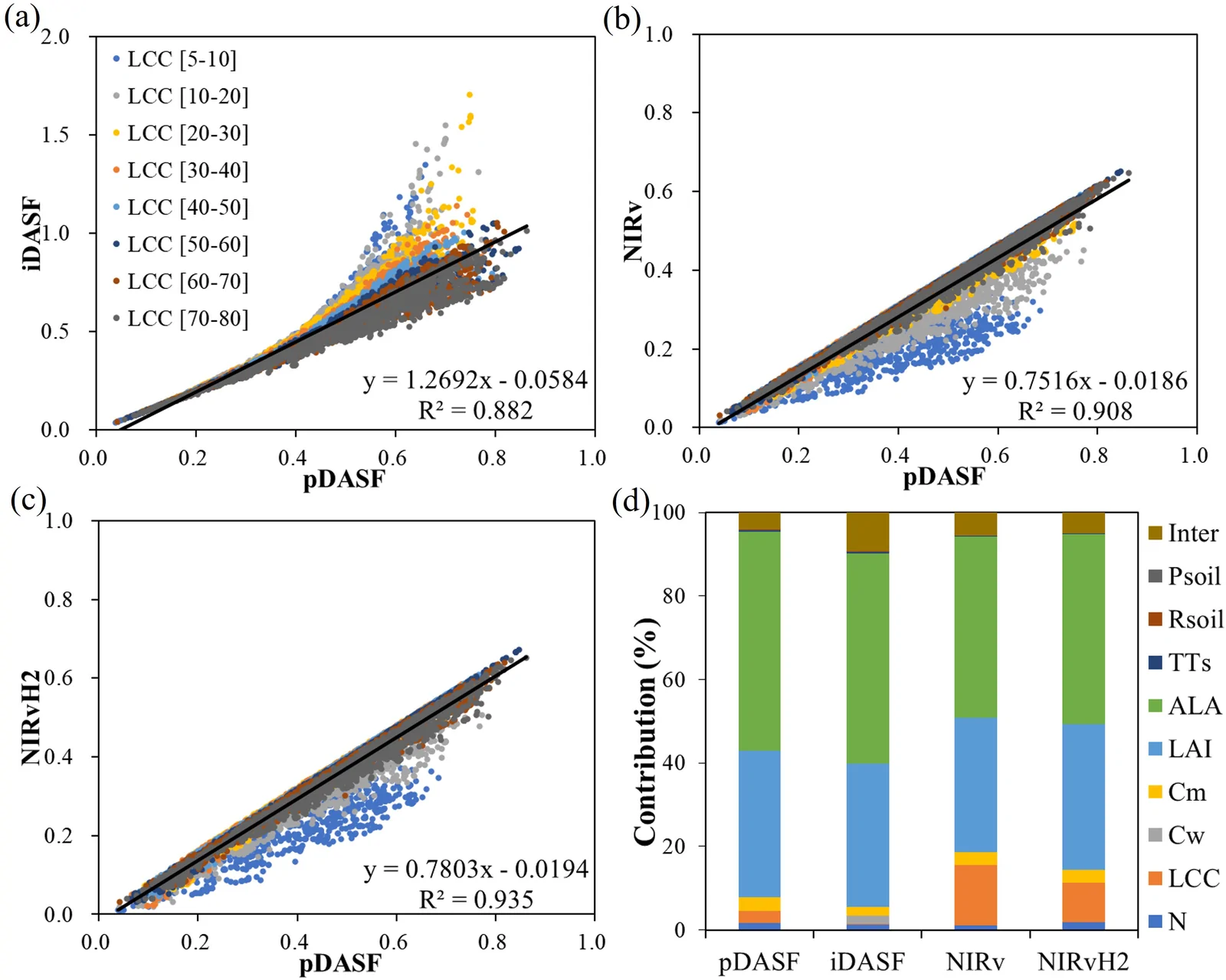
The simulated relationships between pDASF and iDASF **(A)**, NIRv **(B)**, and NIRvH2 **(C)** and the contribution (measured by the first-order sensitivity indices) of PROSAIL-5 parameters to the four DASF proxies using global sensitivity analysis **(D)**. The solid black line in **(A)**, **(B)**, and **(C)** represents the linear regression line fitted to all data points.

### 3.4 Validation of LCC estimation from ground measurements

**Figure 5** demonstrates the measured LCC and the LCC predicted by the RF model using the canopy BRF and CSCs from the Karlsruhe dataset. In general, CSCs outperformed canopy BRF in LCC estimation, with BRF/NIRvH2 achieving the best performance (R²=0.731, RMSE = 5.67 μg/cm², MAE = 4.39 μg/cm²). followed by BRF/NIRv, BRF/pDASF, and BRF/iDASF. Compared to BRF, the RMSE values were reduced by 15.88%, 11.62%, 16.47%, and 16.62% for BRF/pDASF, BRF/iDASF, BRF/NIRv, and BRF/NIRvH2, respectively.

**Figure 5:**
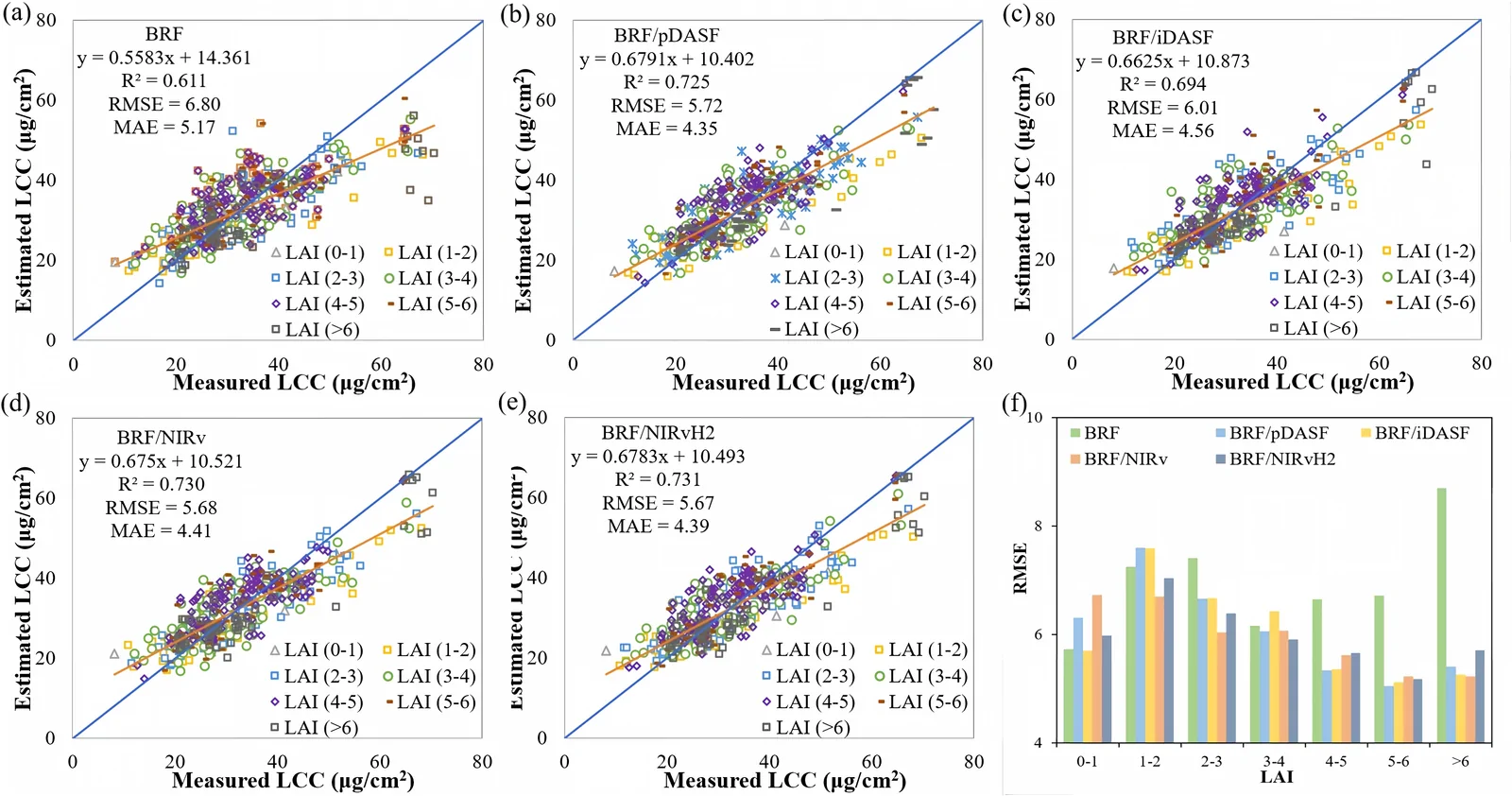
Scatter plots of measured LCC and estimated LCC using ground-measured hyperspectral BRF **(A)**, BRF/pDASF **(B)**, BRF/iDASF **(C)**, BRF/NIRv **(D)**, and BRF/NIRvH2 **(E)**, and RMSE of estimated LCC under different LAI conditions **(F)** in Karlsruhe site.

**Figure 5F** presents the RMSE variations of the five prediction models under various LAI conditions. With LAI increase, the RMSE of canopy BRF predicted LCC firstly decreased then increased, while the RMSE of LCC predicted by CSCs showed a decreasing trend. The improvement of LCC estimation using CSCs in dense canopies is greater than in sparse canopies. The greatest improvement was observed in LAI > 6.0 m²/m², the RMSE values were reduced by 37.87%, 39.60%, 39.94%, and 34.36% for BRF/pDASF, BRF/iDASF, BRF/NIRv, and BRF/NIRvH2, respectively. For LAI < 1.0 m²/m², BRF/iDASF performed similarly with BRF, while BRF/NIRv performed the worst. This may be attributable to limited sample size (n=17). For LAI between 1.0 m²/m² and 2.0 m²/m², BRF/NIRv and BRF/NIRvH2 demonstrated superior accuracy and was more accurate than BRF, BRF/pDASF and BRF/iDASF performed worse than canopy BRF. For LAI > 2.0 m²/m², DASF corrections generally outperformed canopy BRF except for BRF/iDASF in LAI between 3.0 m²/m² and 4.0 m²/m². Notably, BRF/NIRv and BRF/NIRvH2 outperformed BRF/pDASF and BRF/iDASF in LAI between 2.0 m²/m² and 4.0 m²/m² but showed relative declines in LAI between 4.0 m²/m² and 6.0 m²/m².

**Figure 6** compares LCC validation results at the Neusling site, where all samples exhibited LAI values exceeding 2.0 m²/m². Overall, BRF/pDASF achieved the highest accuracy with R² of 0.55, RMSE of 4.74 μg/cm², and MAE of 3.88 μg/cm², followed by BRF/NIRvH2 and BRF/NIRv, while BRF/iDASF slightly outperformed BRF (R² of 0.428, RMSE of 5.32 μg/cm², and MAE of 4.26 μg/cm²). Compared to BRF, the RMSE values were reduced by 10.90%, 0.38%, 8.27%, and 9.21% for BRF/pDASF, BRF/iDASF, BRF/NIRv, and BRF/NIRvH2, respectively.

**Figure 6:**
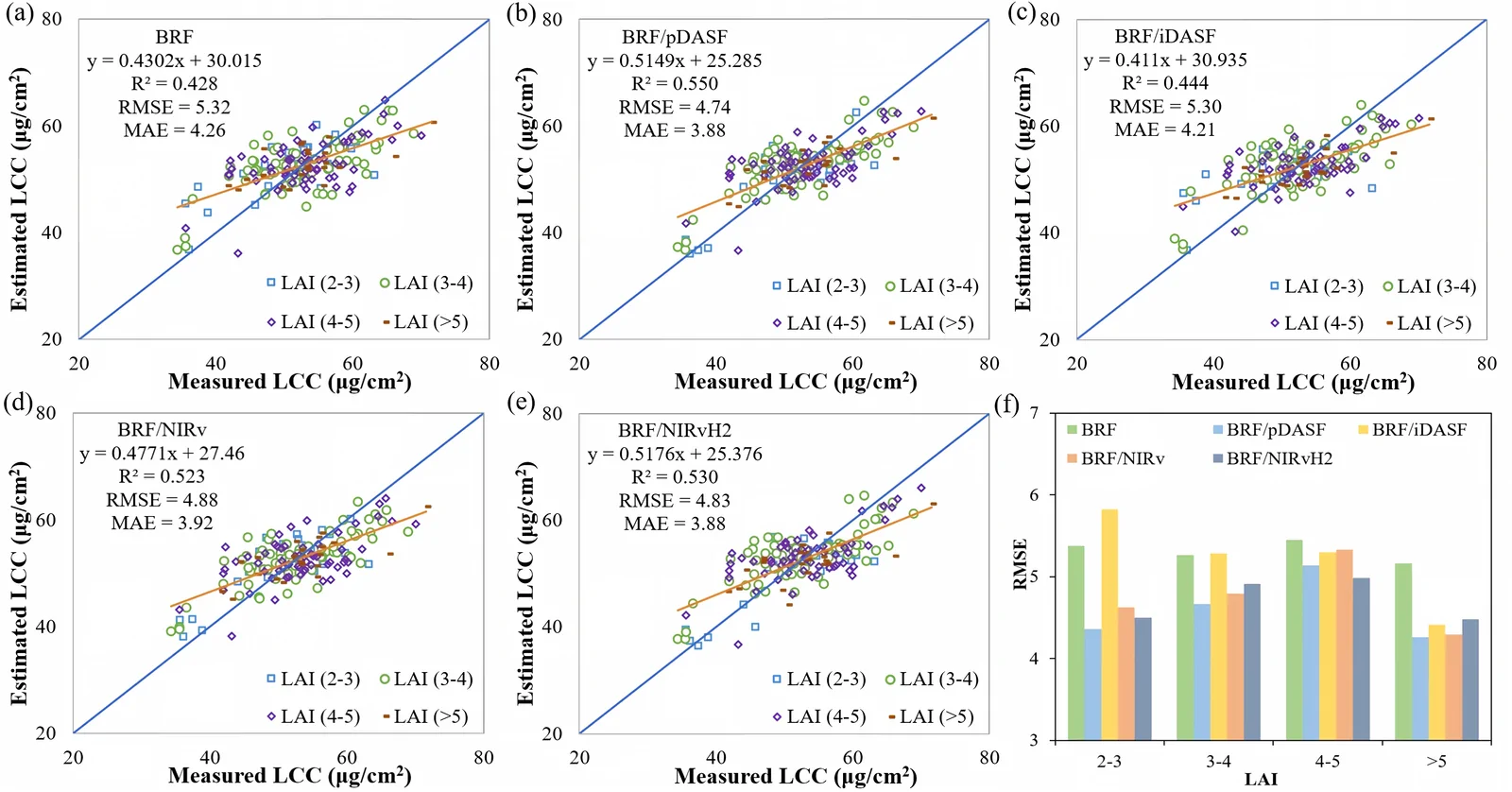
Scatter plots of measured LCC and estimated LCC using ground-measured hyperspectral BRF **(A)**, BRF/pDASF **(B)**, BRF/iDASF **(C)**, BRF/NIRv **(D)**, and BRF/NIRvH2 **(E)**, and RMSE of estimated LCC under different LAI conditions **(F)** in Neusling site.

For different LAI conditions (**Figure 6F**), BRF/pDASF, BRF/NIRv, and BRF/NIRvH2 all achieved higher accuracy than BRF across all LAI ranges, BRF/iDASF outperformed BRF only at LAI >4.0 m²/m². BRF/pDASF achieved the best performance at LAI < 4.0 m²/m² and LAI > 5.0 m²/m², while BRF/NIRvH2 performed the best with LAI range of 4–5 m²/m², BRF/NIRv exhibited lower accuracy than BRF/pDASF across all LAI ranges.

### 3.5 Validation of LCC estimation from simulated Sentinel-2 MSI data

**Figure 7** presents LCC validation results derived from simulated Sentinel-2 MSI data for the Karlsruhe site. Overall, BRF/NIRvH2 achieved the highest accuracy (R² = 0.712, RMSE = 5.84 μg/cm², MAE = 4.53 μg/cm²), corresponding to a 15.97% RMSE reduction relative to uncorrected BRF. Followed by BRF/NIRv, BRF/pDASF, and BRF/iDASF, which also outperformed BRF, with RMSE reductions of 14.36%, 13.29%, and 9.82%, respectively. Across LAI conditions (**Figure 7F**), BRF/NIRvH2 performed the best for sparse canopies (LAI < 2.0 m²/m²), while all the four CSCs showed comparable performance and outperformed BRF for dense canopies (LAI > 3.0 m²/m²).

**Figure 7:**
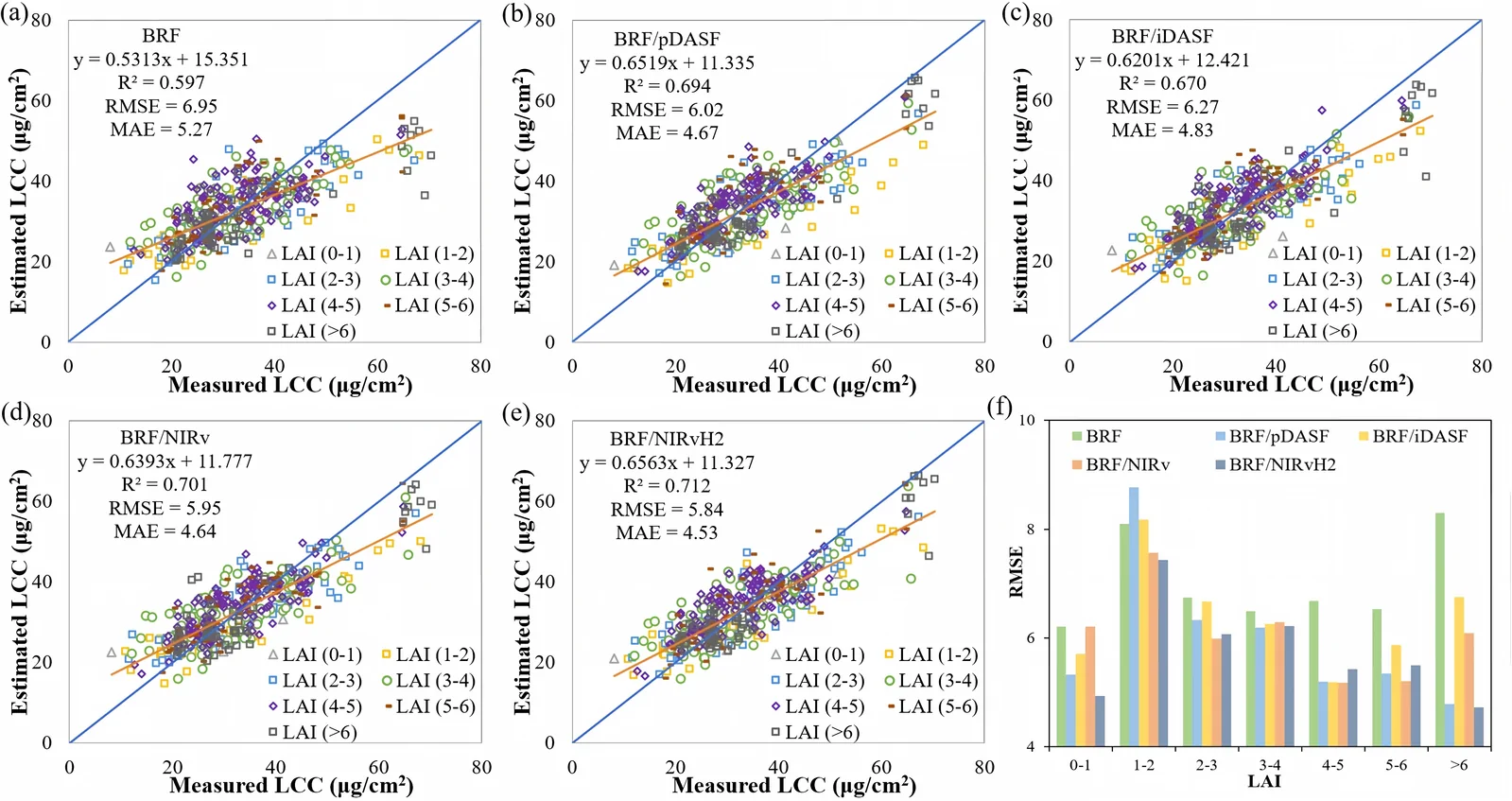
Scatter plots of measured LCC and estimated LCC using simulated Sentinel-2 BRF **(A)**, BRF/pDASF **(B)**, BRF/iDASF **(C)**, BRF/NIRv **(D)**, and BRF/NIRvH2, **(E)** and RMSE of estimated LCC under different LAI conditions**(F)** in Karlsruhe site.

**Figure 8** shows corresponding validation results for the Neusling site. BRF/pDASF delivered the highest accuracy, followed by BRF/NIRvH2, BRF/NIRv, and BRF/iDASF, all showed a better performance than BRF. Compared to BRF, the RMSE values were reduced by 13.09%, 7.44%, 6.05%, and 4.08%, respectively. For different LAI conditions (**Figure 8F**), BRF/pDASF achieved the best performance in LAI < 3.0 m²/m² and LAI > 4.0 m²/m², BRF/NIRv exhibited lower accuracy than BRF/pDASF across all LAI ranges, and underperformed BRF/NIRvH2 at LAI < 4.0 m²/m² but outperformed BRF/NIRvH2 at LAI > 4.0 m²/m².

**Figure 8:**
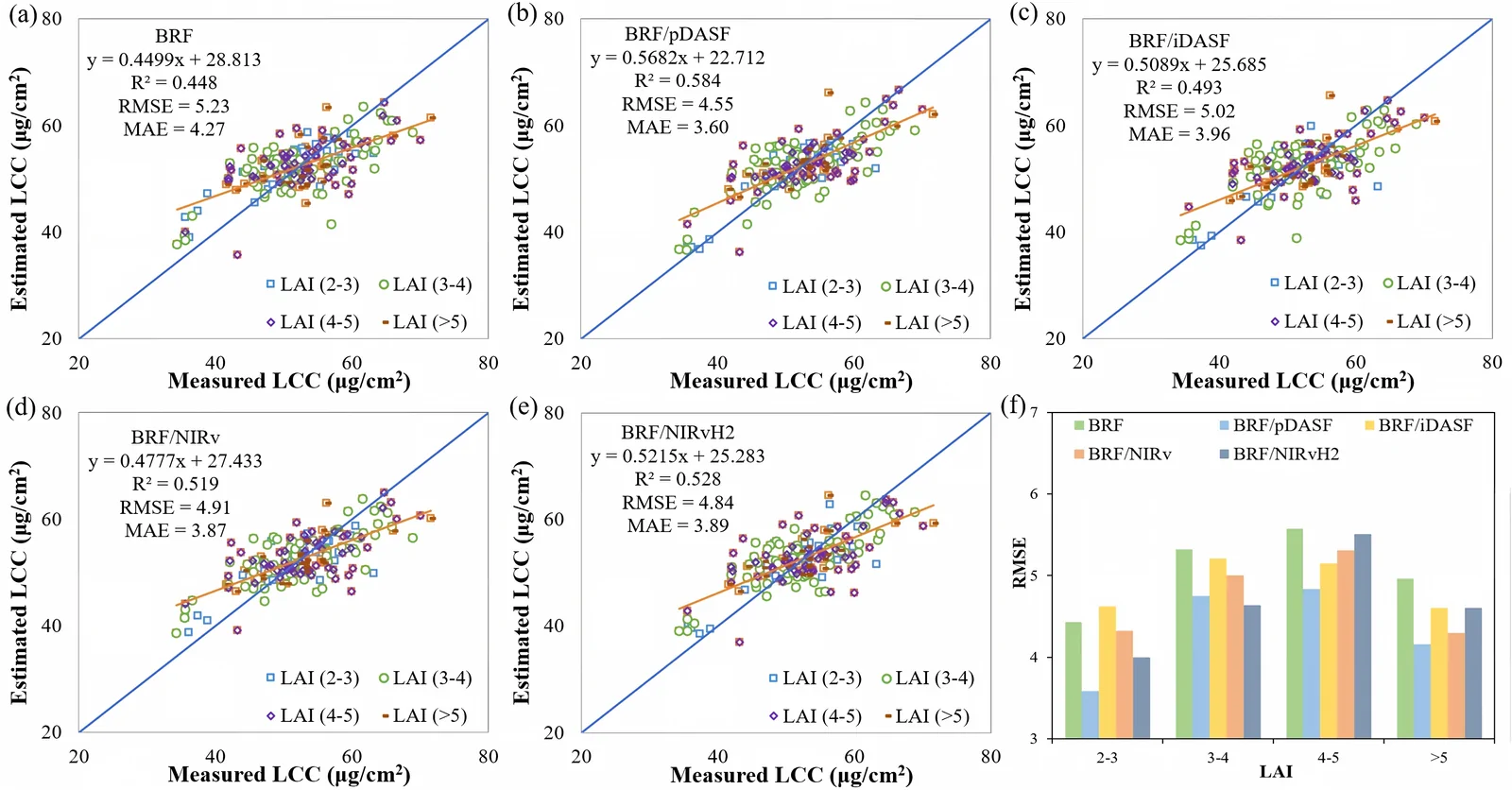
Scatter plots of measured LCC and estimated LCC using simulated Sentinel-2 BRF **(A)**, BRF/pDASF **(B)**, BRF/iDASF **(C)**, BRF/NIRv **(D)**, and BRF/NIRvH2, **(E)** and RMSE of estimated LCC under different LAI conditions **(F)** in Neusling site.

## 4 Discussion

### 4.1 Performance of the four DASF proxies in correcting canopy structural and soil background effect for LCC estimation

Our simulations and measurements demonstrate that DASF-corrected BRF effectively suppresses canopy structural and soil effects while enhancing spectral sensitivity to LCC. Notably, we observed a “red shift” (towards longer wavelengths) in chlorophyll-sensitive spectral peaks within the red-edge region from 691 nm (canopy BRF) to 714–718 nm (CSCs) across both simulated and ground-measured spectra (**Supplementary Figures S8, 9**; **Figure 9**). This spectral shift is attributed to the removal of multiple scattering contributions within the canopy during DASF correction, as BRF is converted to CSC. Multiple scattering is particularly pronounced in the near-infrared and red-edge regions, and its removal allows leaf biochemical absorption features to emerge more clearly. At the canopy scale, the red-edge position is modulated by both leaf chlorophyll content and structural parameters such as LAI and leaf angle distribution; increases in LAI enhance multiple scattering and tend to shift the red edge toward shorter wavelengths. By suppressing this structure-induced “blue shift,” DASF correction enables CSC spectra to more closely reflect leaf-scale biochemical signals. Our variable importance analysis further confirmed this, with the most important spectral bands for LCC prediction shifted from 695–705 nm (canopy BRF) to 700–720 nm (iDASF-, NIRv-, and NIRvH_2_-corrected BRFs) and 770–780 nm (pDASF-corrected BRFs). Conversely, red bands (590–640 nm), which contributed significantly to canopy BRF predictions, showed negligible importance for CSCs, indicating that DASF correction effectively reduces the contribution of spectral regions dominated by structural and soil signals rather than biochemical information.

**Figure 9:**
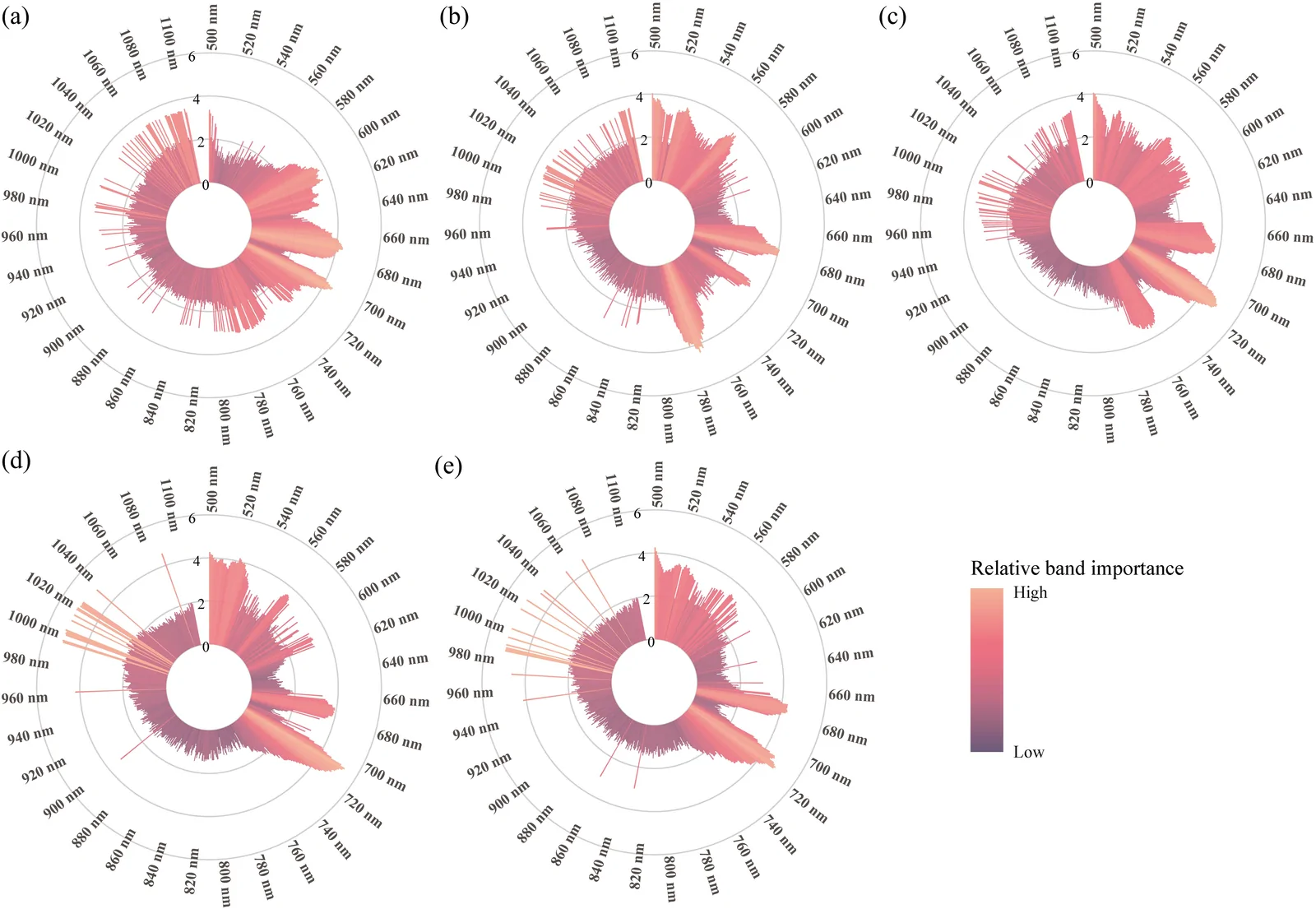
Relative band importance for LCC prediction using ground-measured hyperspectral BRF **(A)**, BRF/pDASF **(B)**, BRF/iDASF **(C)**, BRF/NIRv **(D)**, and BRF/NIRvH2 **(E)** in Karlsruhe site. The numbers outside the circular are the band wavelengths, the numbers on the loop are the relative importance values derived from Boruta algorithm. Higher value indicates greater importance.

The pDASF is efficient but relies on the assumptions of zero soil contribution and negligible leaf absorption to canopy BRF. These constraints limit its applicability in sparse vegetation (Zeng et al., 2018; Liu et al., 2023). Our simulations revealed that pDASF is sensitive to spectral variations associated with soil properties (**Figure 2**; **Supplementary Figures S2-S6**), and in sparse vegetation with smaller LAI, changes in soil reflectance led to greater variation in pDASF (**Supplementary Figure S7**). However, for dense canopies (LAI > 3 m²/m²), pDASF demonstrates reliable performance, which effectively corrected structural effect for LCC estimation. The iDASF accounts for the variation of LMA that neglected by pDASF through incorporating a bias correction factor E. However, the development of iDASF is still under the assumption of black soil, the correcting factor E is estimated and validated under forest scenarios, which may not applicable to sparse herbaceous canopies. Notably, even iDASF demonstrates strong correlation with pDASF (R²=0.88) (**Figure 4A**), it shows scattered relationships at high pDASF (>0.4), and applying iDASF to correct canopy structural effect for LCC estimation showed a worse performance than pDASF, NIRv, and NIRvH2 (**Figures 5**–**8**).

Both NIRv and NIRvH2 exhibit strong linear relationships with pDASF (R²>0.9) and superior LCC estimation capabilities, suggesting that they are good proxies of DASF (Zeng et al., 2018; Liu et al., 2023; Wan et al., 2024). However, at low LCC values (< 40 μg/cm²), the pDASF and NIRv (NIRvH2) relationships were slightly worse, which may attribute to the different sensitivities of NIRv (NIRvH2) and pDASF to LCC (**Figure 2A**). NIRv (NIRvH2) demonstrated enhanced sensitivity to low LCC (< 40 μg/cm²) and but exhibited saturation at high LCC values, which can significantly retaining sensitivity to high LCC values by applying NIRv (NIRvH2) corrections (Wan et al., 2024). In contrast, pDASF displayed a positive correlation with LCC increased from 5 μg/cm² to 80 μg/cm², indicating greater LCC dependence than NIRv (NIRvH2), thus applying pDASF-corrected BRF may remove some sensitivity to high LCC values (Wan et al., 2024). Moreover, (Badgley et al., 2017) and (Zeng et al., 2018) demonstrated that NIRv can reduce a major portion of the soil impact in the total NIR reflectance of the pixel, with NDVI serves as an adjusting factor to represent vegetation fraction (ideally ranges from 0 to 1). Our simulations observed reduced sensitivity of NIRv to soil property variations compared to pDASF (**Figure 2**; **Supplementary Figure S7**). NIRvH2 further minimized the soil effect by using the spectral shape variations in the red-edge region to disentangling the individual contributions from vegetation and soil in the red and NIR, our simulations show NIRvH2 correlates more strongly with pDASF and exhibits lower sensitive to soil background properties than NIRv (**Supplementary Figure S7**). Thus, in our evaluation, both NIRv and NIRvH2 demonstrated relatively higher accuracy compared to pDASF for LCC estimation in sparse vegetation areas (LAI < 2 m²/m²), suggesting that NDVI-based proxies may be more effective under these conditions (**Figures 4**–**6**). However, the relative performance of these proxies may depend on vegetation type, soil properties, canopy structure, and sensor spectral configurations that differ from those considered in our study.

Despite the relative advantages of NIRv and NIRvH2 in sparse canopies, residual soil background effects persist, which is consistent with previous findings. Liu et al. (2023) reported that NIRv fails to adequately capture canopy structural variations when LAI <1 m²/m². The reason may due to that soil NDVI is larger than zero in reality, and NDVI for dense canopy is smaller than one (Zeng et al., 2021). We also found a large variation of NIRvH2 and NIRv corrected BRFs under soil variation in sparse canopies (**Supplementary Figure S7**), underscoring that caution is still needed when applying these proxies under extremely sparse conditions.

### 4.2 Potential applications of DASF estimation methods for satellite platforms

The applicability of the four DASF proxies for correcting canopy structural effects in broadband LCC retrieval was evaluated using simulated Sentinel-2A MSI data derived from field hyperspectral measurements. Our comparative analysis reveals that NIRv and NIRvH2 are superior in canopy structural effect correction and LCC estimation among the four DASF estimation methods. Compared to NIRv, NIRvH2 requires BRF measurement at 678 nm which is the central wavelength of the chlorophyll absorption peak, and at least two red (675–681 nm) or NIR (778–800 nm) bands to derive slope k in Equtaion 8 (Zeng et al., 2021), which is not applicable for most existing multispectral sensors such as MODIS and Landsat-8. However, our results indicated that NIRvH2 can be reliably calculated from Sentinel-2 despite the center wavelength of red band (665 nm) is deviated from 678 nm and the NIR bands exceeding 800 nm, and Sentinel-2 NIRvH2 corrected BRF also demonstrated a robust performance in reducing canopy structure and soil impacts for LCC estimation especially for sparse canopies.

Although pDASF performed poorly in sparse vegetation, it achieved reliable accuracy in dense canopies. However, according to the method in Knyazikhin et al. (2013), pDASF estimation requires at least 2 red edge bands at 710–790 nm. This provides support for its application to Sentinel-2 data, which have three wavebands (704 nm, 740 nm, 782 nm) that can be used to estimate pDASF. Our study and Wan et al. (2024) observed comparable LCC estimation performance between pDASF and NIRv using Sentinel-2 data. Nevertheless, most multispectral UAV and satellite platforms (such as Landsat-8) lack sufficient red-edge bands for pDASF implementation, which constrained its application in these platforms. Compared to pDASF and NIRvH2, NIRV requires only red and NIR bands, which are available in most remote sensing platforms, showed higher potential and applicability for LCC estimation across different remote sensing platforms. We observed a better performance of BRF/NIRv than canopy BRF using simulated Landsat-8 data (**Supplementary Figure S10**).

### 4.3 Application prospects and future work

The results presented in this study, which leverages operational satellite data (e.g., Sentinel-2) and robust machine learning models, holds significant promise for a range of practical applications. In precision agriculture, the developed LCC retrieval algorithm can be operationalized to generate field-scale chlorophyll maps. These maps can guide variable-rate nitrogen fertilization, helping farmers optimize resource use, reduce environmental pollution, and enhance crop yields. At a regional scale, our approach facilitates the monitoring of vegetation health and productivity over large areas, providing critical data for ecosystem management and climate change impact studies.

Compared with previous studies that have employed DASF-based proxies for vegetation biochemical estimation, our work provides the first systematic comparison of four DASF proxies under varying canopy conditions and sensor configurations. For instance, while pDASF has been successfully applied to forest canopies (Knyazikhin et al., 2013) and iDASF has shown improved performance in dense agricultural canopies (Lin et al., 2023), their comparative efficacy in sparse canopies and across different sensors has remained largely unexplored. Our results demonstrate that spectral-index-based proxies (NIRv and NIRvH_2_) offer practical advantages in sparse canopies due to their operational simplicity, whereas radiative-transfer-based proxies (pDASF and iDASF) excel in dense canopies where soil effects are minimal. This differential applicability aligns with the findings of Zeng et al. (2021) regarding the residual soil sensitivity of NIRv, while extending their analysis to a direct comparison with pDASF and iDASF. Furthermore, the consistent improvement of DASF-corrected BRF over uncorrected BRF (RMSE reductions of 6.05%–16.62%) corroborates the theoretical framework of spectral invariants theory and extends its practical utility from forest ecosystems to agricultural systems, which has been previously demonstrated in the context of sun-induced chlorophyll fluorescence (SIF) (Badgley et al., 2017; Zeng et al., 2018) but not systematically for LCC retrieval. Moreover, while Zeng et al. (2021) suggested that NIRvH2 substantially minimizes soil background effects, our findings indicate that residual soil influences persist, particularly under extremely sparse conditions.

However, the generalizability of this study is also subject to several limitations that need to be further investigated. Specifically, the limited availability of *in situ* measurements across a representative range of leaf area index (LAI) conditions introduce uncertainty. For instance, the Neusling dataset only includes LAI values above 2.0 m²/m², potentially influencing validation accuracy under sparse vegetation conditions. Moreover, our LCC estimations rely on simulated Sentinel-2 and Landsat-8 observations, which may not fully capture the complexities of actual satellite retrievals, such as atmospheric perturbations, viewing geometry variations, and radiometric calibration uncertainties. Previous studies have demonstrated that these factors can significantly influence vegetation index-based LCC estimates (Verrelst et al., 2015; Berger et al., 2020). To enhance the robustness of our findings, future efforts should prioritize the collection of ground data encompassing a wider spectrum of leaf biochemical and canopy structural traits. Furthermore, evaluating the four DASF estimation methods with actual observations from both existing missions (e.g., MODIS, Sentinel-3) and forthcoming hyperspectral sensors (e.g., FLEX, DESIS, EnMAP) is recommended to better assess their utility in mitigating confounding effects in LCC retrieval.

An additional limitation concerns the biogeographical scope of the validation data, which were derived from mid-latitude agricultural and grassland systems and acquired using heterogeneous field protocols. The relative performance of these proxies may vary with vegetation type, soil properties, and canopy architecture, since their effectiveness in mitigating soil effects depends on the spectral contrast between vegetation and soil. Future work should therefore expand validation efforts to include diverse soil backgrounds (e.g., varying in type, organic carbon content, and surface moisture) and a wider range of climate zones (e.g., arid, tropical). Besides, non-photosynthetic vegetation (NPV) constitutes another significant confounding factor. Previous studies indicate that NPV can substantially modify canopy BRF and vegetation index signals, with its effect being highly dependent on cover fraction, moisture status, and stage of decomposition (Gao et al., 2022). Neglecting NPV variability may thus lead to biased LCC estimates and weakened CSC–LCC relationships. It is therefore critical that subsequent research explicitly incorporates NPV as an auxiliary variable to improve the physicochemical interpretability and accuracy of LCC retrieval models. Future work will focus on upscaling the algorithm and validating it with comprehensive datasets and multi-source satellite imagery (e.g., Landsat, MODIS) to support global-scale vegetation biochemical monitoring.

## 5 Conclusions

This study systematically evaluates four DASF estimation methods to decouple canopy structural effect on canopy BRF for LCC estimation across agricultural canopies. Our results demonstrated that DASF could well characterize the effect of canopy structure from canopy BRF and enhance spectral sensitivity to LCC. LCC retrieval models were validated against field measurements from both GFZ and EcoSIS datasets, the results showed that correcting BRF by DASF improved LCC retrieval with RMSE values reduced by 0.38–16.62% compared to canopy BRF for ground, simulated Sentinel-2 data. NIRv and NIRvH2 demonstrate relatively higher accuracy in sparse canopies compared to pDASF and iDASF, likely due to their partial mitigation of soil background effect, while pDASF showed reliable performance in dense vegetation. Crucially, NIRv is simpler to calculated, shows great potential for multi-sensor applications. However, residual soil impacts on NIRv and NIRvH2 still exist in sparse canopies (LAI < 1 m²/m²). These findings underscore the significant potential of DASF corrections for operational LCC monitoring. Future work with comprehensive datasets to refine these methods for diverse vegetation conditions, especially sparse canopies, will further enhancing their usefulness in comprehensive vegetation biochemical assessments across various ecosystems.

## Data Availability

The original contributions presented in the study are included in the article/**Supplementary Material**. Further inquiries can be directed to the corresponding author.
