# Supplementary material for: The performance of directional area scattering factor proxies for correcting canopy structural and soil effects on leaf chlorophyll content estimation varies with canopy conditions in agricultural systems
Source: Front Plant Sci. 2026 Sep 30;17:1884277. doi: 10.3389/fpls.2026.1884277 (PMC13661556; doi:10.3389/fpls.2026.1884277)
Supplement: Supplementary file 1 [file DataSheet1.doc]

Supplementary material

# 1 Supplementary Data

**1.1 Global sensitivity analysis**

In this study, we employed the Sobol’ method (Sobol', 2001) , implemented in the Python ‘SALib’ package (Herman and Usher, 2017) , to conduct GSA on the PROSAIL simulated datasets. The Sobol' method, a variance-based GSA approach, is particularly effective in quantifying both main sensitivity effects (Si, or first-order effects), which represent the individual contribution of each input parameter to the model output, and total sensitivity effects (STi), which encompass the first-order effects along with interactions between input variables, thereby capturing the overall contribution of each parameter to the model output (Song et al., 2012). Key parameter configurations used in this study include: Saltelli sampling scheme, a sample size of 1024 per parameter, 100 bootstrap resamples for uncertainty quantification, and a 95% confidence interval level. Both sensitivity indices are mathematically expressed as follows:

 (1)

 (2)

 (3)

where Vi (Vi = V[E(Y|xi)]) represents the first-order variance for each input factor; V is the attribution of total output variance; and Vij (Vij = V[E(Y|xi, xj)] - Vi - Vj) to V1,…,k represents the interactions among k factors.

**1.2 Soil spectral reflectance representing various soil types and soil moisture**

Soil spectral reflectance data for various soil types were obtained from the dataset of (Liu et al., 2002), which includes 92 globally collected soil samples representing a wide range of soil types. The soil samples were air-dried to zero volumetric soil moisture content (SMCv) to avoid the influence of soil moisture, spectral reflectance over the 350–2500nm wavelength region were acquired with a full-range (350–2500 nm) ASD Pro FR portable spectroradiometer (Analytical Spectral Devices, Boulder, CO). To avoid data redundancy caused by similar soil spectra, we used the spectral angle mapping classification method (Kruse et al., 1993) to identify representative spectra. The criterion with a spectral angle smaller than 0.05 between two different spectra was used to identified similar reflectance (Gao et al., 2022; Jia et al., 2016). Finally, 31 spectral reflectance curves representing different soil types were kept for PROSAIL simulation (Supplementary Figure 1a).

Soil spectral reflectance at 14 volumetric soil moisture content (SMCv) levels (ranges from 0 to 70%) was obtained from the dataset of (Lobell and Asner, 2002). The soil sample, classified as Mollisol, was collected from a temperate savanna field in Texas, USA. Reflectance measurements were conducted in a laboratory using ASD Pro FR spectroradiometer. After measuring the reflectance of the oven-dried sample, deionized water was incrementally added until the soil reached near saturation. Reflectance measurements were repeatedly collected as the soil dried back to its initial mass (Supplementary Figure 1b).

# 1.3 Validation of LCC estimation from simulated Landsat-8 OLI data

Due to the lack of spectral bands required for calculating pDASF, iDASF, and NIRvH2, we only calculated NIRv from simulated Landsat-8. Overall, the accuracy of LCC estimated from simulated Landsat-8 data was lower than hyperspectral and Sentinel-2 data. BRF/NIRv showed a better performance than canopy BRF, with RMSE values reduced by 7.97% and 0.81%, respectively for Karlsruhe and Neusling site (Supplementary Figure 10).

(a)

(b)

**Supplementary Figure 1.** Reflectance spectra of 31 soil types (a) and 14 volumetric soil moisture content (SMCv) levels (b) used as input into the PROSAIL model.

**Supplementary Figure 2.** Simulated sensitivities of BRF, BRF/pDASF, BRF/iDASF, BRF/NIRv, and BRF/NIRvH2 to the variations in soil type (a), soil moisture content (b), and soil brightness (c) using PROSAIL-5 model. For each simulation, only one parameter was changed with the other parameters default. The simulated BRF and DASF-corrected BRFs were under LAI=0.5 m2/m2. SMCv represents volumetric soil moisture content.

**Supplementary Figure 3.** Simulated sensitivities of BRF, BRF/pDASF, BRF/iDASF, BRF/NIRv, and BRF/NIRvH2 to the variations in soil type (a), soil moisture content (b), and soil brightness (c) using PROSAIL-5 model. For each simulation, only one parameter was changed with the other parameters default. The simulated BRF and DASF-corrected BRFs were under LAI=1.0 m2/m2. SMCv represents volumetric soil moisture content.

**Supplementary Figure 4.** Simulated sensitivities of BRF, BRF/pDASF, BRF/iDASF, BRF/NIRv, and BRF/NIRvH2 to the variations in soil type (a), soil moisture content (b), and soil brightness (c) using PROSAIL-5 model. For each simulation, only one parameter was changed with the other parameters default. The simulated BRF and DASF-corrected BRFs were under LAI=3.0 m2/m2. SMCv represents volumetric soil moisture content.

**Supplementary Figure 5.** Simulated sensitivities of BRF, BRF/pDASF, BRF/iDASF, BRF/NIRv, and BRF/NIRvH2 to the variations in soil type (a), soil moisture content (b), and soil brightness (c) using PROSAIL-5 model. For each simulation, only one parameter was changed with the other parameters default. The simulated BRF and DASF-corrected BRFs were under LAI=4.0 m2/m2. SMCv represents volumetric soil moisture content.

**Supplementary Figure 6.** Simulated sensitivities of BRF, BRF/pDASF, BRF/iDASF, BRF/NIRv, and BRF/NIRvH2 to the variations in soil type (a), soil moisture content (b), and soil brightness (c) using PROSAIL-5 model. For each simulation, only one parameter was changed with the other parameters default. The simulated BRF and DASF-corrected BRFs were under LAI=6.0 m2/m2. SMCv represents volumetric soil moisture content.

**Supplementary Figure 7.** Simulated sensitivities of pDASF, iDASF, NIRv, and NIRvH2 to the variations in soil type, soil moisture content, and soil brightness under different LAI conditions using PROSAIL-5 model.

**Supplementary Figure 8.** GSA first-order sensitivity indices (Si) of PROSAIL-5 parameters to BRF (a), BRF/pDASF (b), BRF/iDASF (c), BRF/NIRv (d), and BRF/NIRvH2 (e).

**Supplementary Figure 9.** Circular bar plot of the spectral band relative importance for LCC prediction using ground-measured hyperspectral BRF (a), BRF/pDASF (b), BRF/iDASF (c), BRF/NIRv (d), and BRF/NIRvH2 (e) in Neusling site. The number outside the circular is the band wavelength, the number on the loop is the relative importance value derived from Boruta algorithm, the higher the value, more importance of the band.

**Supplementary Figure 10.** Scatter plots of measured LCC and estimated LCC using simulated Landsat-8 BRF (a) and NIRv (b) in Karlsruhe site and simulated Landsat-8 BRF (c) and BRF/NIRv (d) in Neusling site.

# References

Gao L, Darvishzadeh R, Somers B, Johnson BA, Wang Y, Verrelst J, et al. Hyperspectral response of agronomic variables to background optical variability: Results of a numerical experiment. Agricultural and Forest Meteorology 2022; 326:109178.

Herman J, Usher W. SALib: An open-source Python library for Sensitivity Analysis. The Journal of Open Source Software 2017; 2.

Jia K, Liang S, Gu X, Baret F, Wei X, Wang X, et al. Fractional vegetation cover estimation algorithm for Chinese GF-1 wide field view data. Remote Sensing of Environment 2016; 177: 184-191.

Kruse FA, Lefkoff AB, Boardman JW, Heidebrecht KB, Shapiro AT, Barloon PJ, et al. The spectral image processing system (SIPS)—interactive visualization and analysis of imaging spectrometer data. Remote Sensing of Environment 1993; 44: 145-163.

Liu W, Baret F, Gu X, Tong Q, Zheng L, Zhang B. Relating soil surface moisture to reflectance. Remote Sensing of Environment 2002; 81: 238-246.

Lobell DB, Asner GP. Moisture Effects on Soil Reflectance. Soil Science Society of America Journal 2002; 66: 722-727.

Sobol' IM. Global sensitivity indices for nonlinear mathematical models and their Monte Carlo estimates. Mathematics and Computers in Simulation 2001; 55: 271-280.

Song X, Bryan BA, Paul KI, Zhao G. Variance-based sensitivity analysis of a forest growth model. Ecological Modelling 2012; 247: 135-143.
